# Life after Harvest: Circadian Regulation in Photosynthetic Pigments of Rocket Leaves during Supermarket Storage Affects the Nutritional Quality

**DOI:** 10.3390/nu11071519

**Published:** 2019-07-04

**Authors:** Lorena Ruiz de Larrinaga, Victor Resco de Dios, Dmitri Fabrikov, José Luis Guil-Guerrero, José María Becerril, José Ignacio García-Plazaola, Raquel Esteban

**Affiliations:** 1 Department of Plant Biology and Ecology. University of the Basque Country (UPV/EHU), 48080 Bilbao, Spain; 2 Department of Crop and Forest Sciences & AGROTECNIO Center, University of Lleida, 25198 Lleida, Spain; 3 Food Technology Division, University of Almería, CeiA3, 40120 Almería, Spain; 4 Basque Centre for Climate Change (BC3), 48640 Leioa, Spain

**Keywords:** carotenoids, circadian clock, light, optical indices, supermarket, zeaxanthin

## Abstract

Vegetables, once harvested and stored on supermarket shelves, continue to perform biochemical adjustments due to their modular nature and their ability to retain physiological autonomy. They can live after being harvested. In particular, the content of some essential nutraceuticals, such as carotenoids, can be altered in response to environmental or internal stimuli. Therefore, in the present study, we wondered whether endogenous rhythms continue to operate in commercial vegetables and if so, whether vegetable nutritional quality could be altered by such cycles. Our experimental model consisted of rocket leaves entrained under light/darkness cycles of 12/12 h over 3 days, and then we examined free-run oscillations for 2 days under continuous light or continuous darkness, which led to chlorophyll and carotenoid oscillations in both constant conditions. Given the importance of preserving food quality, the existence of such internal rhythms during continuous conditions may open new research perspective in nutrition science. However, while chromatographic techniques employed to determine pigment composition are accurate, they are also time-consuming and expensive. Here we propose for the first time an alternative method to estimate pigment content and the nutritional quality by the use of non-destructive and in situ optical techniques. These results are promising for nutritional quality assessments.

## 1. Introduction

The intake of carotenoids as β-carotene (β-C), lutein (L), and zeaxanthin (Z) is associated with ocular health [1,2,3] and cognitive benefits [4]. This is probably due to their role as antioxidant agents [5], as has been demonstrated in plant and animal tissues [6]. In addition, a diet rich in carotenoids has been associated with cancer resistance [7,8] and prevention of cardiovascular diseases [9]. Because humans cannot synthesize carotenoids, they should be consumed in the diet. However, the content of some essential carotenoids is extremely variable in edible vegetables because phytochemicals respond to environmental stimuli [10]. In fact, several pre-harvesting, but also post-harvesting factors have been identified as regulators of the carotenoid pool: growing environment and cultivation method, genotype, ripening time, and/or processing conditions [11].

The modular design of plants allows individual organs, such as isolated leaves, to manifest autonomous functions and carry out metabolic processes, such as photosynthesis, respiration or light-dependent biological processes, even after being harvested [12]. That is, they retain the ability to perceive and respond to external stimuli. One of those stimuli is the succession of day (light) and night (dark) cycles, which is able to set internal clocks. Biological circadian clocks seem to be a common feature that has evolved genetic routes capable of maintaining 24-h oscillations. The circadian system affects numerous physiological [13] and molecular processes in plants and enables them to anticipate and predict daily environmental cues, such as dawn [14]. The composition of light-harvesting antennae is also under circadian regulation, as shown by the chlorophyll a to b ratio (Chl a/b) [15], which oscillates depending on light cues.

Considering the case of vegetables stored in supermarket shelves, they may be exposed to different artificial light conditions (and display consequently different nutritional content) based on their position on the shelves (such as continuous illumination if they are located in the front or continuous darkness if they are located at the back). If rhythmicity in pigments (chlorophylls and carotenoids) is maintained after harvest, the nutritional quality of these vegetables could be potentially affected by the position they occupy on the shelves. To the best of our knowledge, only a handful of studies have tested whether 24-h-cycles of light and dark during postharvest storage preserve both the nutritional content and plant tissue integrity [16]. However, whether or not photosynthetic pigments are able to maintain circadian function after harvest has not yet been tested. Consequently, the potential effects of different light regimes on carotenoid-derived nutritional values remain unknown.

Given the importance of the preservation of food quality, the main purpose of this study was to verify that internal rhythms operate in stored vegetables, and in particular, whether pigment composition is under such regulation. We chose to focus on rocket (*Eruca sativa* Mill.) as this is a commercially important crop that has been shown to provide beneficial and health-promoting compounds to the human diet [17]. More specifically, in this work, we aimed to (i) determine whether pigments in harvested vegetables stored in the supermarket show circadian rhythms and to (ii) determine whether these rhythms affect the nutritional content of green leafy vegetables stored on supermarket shelves. Pigments (e.g., violaxanthin+antheraxanthin+Z pool, VAZ) can be easily estimated with non-destructive techniques by reflectance measurements [18,19], such as the photochemical reflectance index (PRI), which is based on reflectance wavelengths at 531 nm and 570 nm. This index is used as a proxy for the changes in the xanthophyll cycle pigment pool at the leaf level (mainly in areas of agronomy and ecology) [18], so, we also aimed (iii) to test whether PRI may be used as a quick, non-destructive proxy for pigment concentration in the supermarket, thus offering the prospect of nutritional quality assessments.

## 2. Materials and Methods

### 2.1. Biological Material, Experimental Design, and Sampling

Rocket leaves packaged in protected atmospheres inside biaxially oriented polypropylene bags were sourced from a local supermarket with at least 15 days until the date of expiry. The packages were stored at 4 °C in dark conditions for 12 h prior to the experiment to assure comparable conditions in the phytochemical profiles. Environmental conditions during entrainment were set to mimic the environment of supermarket shelves. We performed the experiments in a cold chamber with the temperature constant at 4 °C and the relative humidity at 65 ± 4.5%. Radiation was kept at ≈6 μmol m^−2^ s^−1^ of photosynthetically active radiation, (LI-189B, Li-Cor, Lincoln, NE, USA) and provided by LED lamps hung 30 cm above the rocket bags. The experimental design consisted of an entrainment phase (the process in which external cues set the circadian clock [20]) of 3 days with a photoperiod set to 12 h of light and 12 of darkness, followed by continuous conditions (2 days) of darkness or light. The selection of these short times for entrainment and the continuous phase was due to the short lifespan of packaged rocket. Samples were collected every 4 h from the end of the entrainment phase (last 12 h) and until the samples had been exposed to 50 h of continuous conditions (62 h total). In each of the sampling periods, 5 replicates per condition (constant dark or light) were collected for analysis. Each replicate was acquired by a random sampling of twenty different leaves from one independent bag, and afterward, these leaves were uniformly mixed. For the rest of the measurement parameters, 8 replicates per condition (constant dark or light) were performed. Once the bag was opened, it was discarded. This experimental design was repeated twice (with no significant differences between them at *p* > 0.05 after Student’s *t*-test).

### 2.2. Visual/Freshness Quality and Chlorophyll a Fluorescence Determination

The visual/freshness quality of the leaf tissues was evaluated for quality traits, including color and freshness. The visual/freshness evaluation was performed by two independent laboratory personnel using a semi-quantitative scale of 1 to 5. The color scale was: 5, >90% green and fresh appearance; 4, <80% green and fresh appearance; 3, <25% yellowing and not fresh; 2, >25% yellowing and not fresh; 1, >50% yellowing and not fresh. The chlorophyll *a* fluorescence, as an indicator of the functional status of the photosynthetic apparatus [21], was measured with a portable chlorophyll fluorimeter (Fluorpen FP100, PSI, Brno, Czech Republic) on dark-adapted leaves (for 15 min). This period permitted determination of the basal fluorescence (Fo). Subsequently, we determined the maximal fluorescence (Fm) with a saturation pulse (3000 μmol photons m^−2^ s^−1^). The maximal photochemical efficiency of photosystem II was then calculated by the following ratio Fv/Fm = (Fm − Fo)/Fm. Leaves with visual/freshness values lower than 4, and an Fv/Fm lower than 0.6 were not used for further analysis.

### 2.3. Leaf Optical Properties and Index Calculations

Before destructively sampling leaf tissue, leaf optical properties were captured from the adaxial surface of the leaves destined for leaf biochemistry measurements. We measured the leaf PRI with a PRI-meter (PlantPen PRI 200, PSI, Brno, Czech Republic) on the adaxial side of the leaves. This index was obtained by comparing two reflectance wavelength bands (531 nm and 570 nm), and the formula was as follows: (R531 − R570)/(R531 + R570) [18,19]. The normalized difference vegetation index (NDVI), an indicator of chlorophyll content in plants, was measured with an NDVI-meter (PlantPen PRI 200, PSI, Brno, Czech) using the reflectance difference in the visible and the near-infrared wavelengths: (R740 − R660)/(R740 + R660) [22]. The additional chlorophyll content index (CCI) was determined with a CCM-200 chlorophyll content meter (Opti-Sciences, Inc., Hudson, NH, USA) and calculated as the difference between the optical absorbance at 653 nm and 931 nm.

### 2.4. Analysis of Carotenoids and Chlorophylls

Pigments were extracted following the method of Esteban et al. [23]. Briefly, we extracted the plant material using a Tearor 985,370 electric tissue homogenizer (BioSpec, Bartlesville, OK, USA) with 1 mL of acetone (100%) with 0.5 g/L of CaCO_3_ at ≤4 °C using cold racks (IsoPack, Eppendorf IsoTherm^®^, Madrid, Spain) to avoid acid traces that might change pigment composition. Once homogenized, samples were centrifuged at 16,000× *g* for 20 min at 4 °C and syringe-filtered through a 0.22 μm PTFE filter (Teknokroma, Barcelona, Spain). Extracts were injected (15 μL) on a reversed-phase C18 column HPLC system (Waters Spherisorb ODS1, 4.6 × 250 mm, Milford, MA, USA) following the method of Garcia-Plazaola and Becerril [24] with modifications [25,26]. The 717 plus autosampler was equipped with a thermostat that maintained the temperature at a constant 4 °C to avoid pigment degradation. Photosynthetic pigments were measured with a PDA detector (Waters model 996 Milford, MA, USA) in the range 250 to 700 nm. Peaks were detected and integrated at 445 nm for carotenoid and chlorophyll content. Pigments were identified by comparing spectral characteristics obtained with the PDA detector and retention times with those of standard materials (DHI, Hørsholm, Denmark). Pigments were quantified using standard reference curves as previously described [24,26,27]. The total V+A+Z pool was estimated as the sum of violaxanthin, antheraxanthin, and zeaxanthin. The de-epoxidation index (A+Z/V+A+Z) was calculated as the sum of epoxidated xanthophylls (antheraxanthin and zeaxanthin) divided by the total V+A+Z. This index indicates the kinetic changes in the de-epoxidation state of the xanthophyll cycle that directly correlates with the A+Z content. Total carotenoids were calculated as the sum of neoxanthin (N), V, A, L, Z, and β-C. The results are expressed and discussed on a dry weight (DW) basis for chlorophylls (Figure 2b and 6a) VAZ (Figure 6b) and total carotenoids (Figure 6c). For the rest of Figures 3–5 carotenoids are expressed and discussed on a chlorophyll basis (for carotenoids).

### 2.5. Tocopherol, Phytosterol and Fatty Acid Extraction and Analysis

Tocopherols, phytosterols, and fatty acids were extracted and determined by HPLC analysis according to Guil-Guerrero et al. [28]. Prior to analysis of tocopherols and phytosterols, 0.2 g of dry matter sample was saponified following the López–Ortiz method [29]. Tocopherols were determined using an RP-HPLC/DAD (Agilent 174 1100 series, Palo Alto, CA, USA) equipped with a ProntoSIL C30 column (4.6 × 250 mm, 3 μm; Bischoff Chromatography, Atlanta, GA, USA) cooled at 15 °C in the HPLC system (Agilent 1100 series, Palo Alto, CA, USA) according to Pérez-Fernandez et al. [30]. Mixtures of methanol:acetonitrile (95:5, *v*/*v*, phase A) and 2-propanol:n-hexane (50:50, *v*/*v*, phase B) were used as the mobile phase at a flow rate of 0.8 mL/min. The following sequence was used to elute each sample: 25 min of phase A (100%) followed by 20 min of phase B (100%). An additional 15 min of phase A (100%) was used to re-equilibrate the column. Phase B was used as a washing solution. The wavelength selected for DAD was 290 nm. Tocopherols were quantified using α-tocopheryl acetate as external standard for reference curve. Phytosterols were determined using RP-HPLC/DAD with a Luna C18 column (250 × 4.6 mm, 5 μm; Phenomenex, Torrance, CA, USA) at a fixed temperature of 30 °C in the HPLC system (Agilent 1100 series, Palo Alto, CA, USA). The mobile phase was programmed in isocratic mode, containing methanol:acetonitrile (70:30, *v*/*v*), and the flow rate was 0.8 mL/min for 55 min. The wavelength selected for DAD was 210 nm. They were quantified using stigmasterol as external standard for reference curve.

### 2.6. Data Processing and Statistics

**Figure 1:**
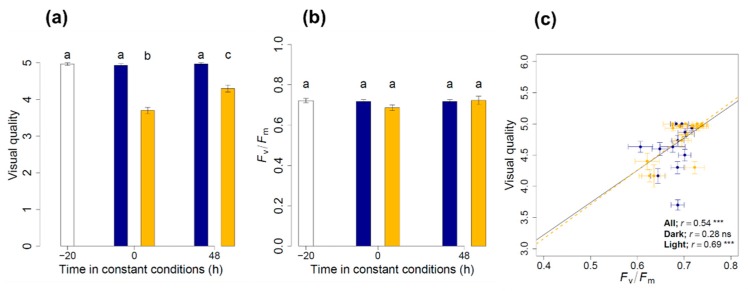
Visual and physiological characterization of rocket. The visual and physiological characterizations were performed by visual quality (**a**) and photochemical efficiency (**b**; Fv/Fm) measurements performed at three different stages of the experiment: at −20 h (beginning of the entrainment phase), 0 h (beginning of the continuous phase) and 48 h in continuous illumination conditions of darkness (blue) and light (yellow). Different lowercase letters indicate significant difference after Tukey’s test (*p* = 0.05). Values are the mean of 10 to 16 replicates ± S.E. Positive correlation between visual quality and photochemical efficiency (Fv/Fm) (panel **c**) throughout the experiment and the continuous phase (yellow, light conditions and blue, dark conditions). Linear correlations are shown when significant at *p* < 0.05 (*** *p* < 0.0001; n.s., not significant).

**Figure 2:**
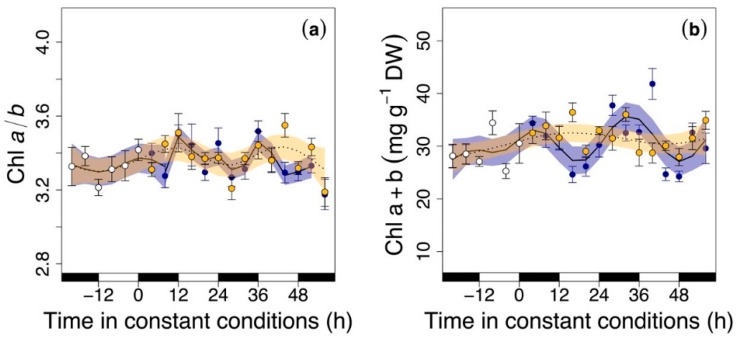
Chlorophylls in rocket leaves during the last cycle of the entrainment phase (white circles) and under continuous darkness (blue circles) and light (yellow circles): (**a**) the chlorophyll a to b ratio (Chl a/b) and (**b**) total chlorophylls expressed on dry weight (Chl a+b; mg g^−1^ DW). Black and white rectangles on the *x*-axis represent subjective night and day, respectively (that is, when leaves would have experienced nighttime or daytime conditions). Time zero represents the time when constant conditions started. Temporal patterns were examined with the generalized additive model (GAM) fitted with an automated smoothness selection [31]. Shaded areas indicate the 95% confidence interval (CI) of the fitted GAM. Values are the mean of 10 replicates ± standard error (S.E.)

**Figure 3:**
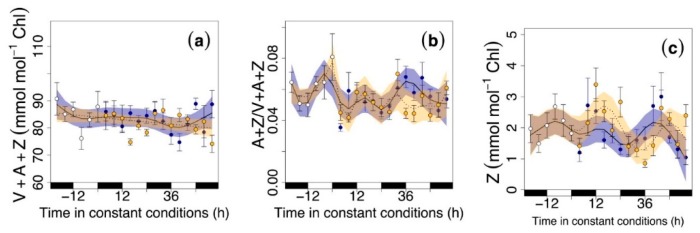
Xanthophylls in rocket leaves during the last cycle of the entrainment period (white circles) and under continuous dark (blue circles) and light conditions (yellow circles): (**a**) the total pool of xanthophyll cycle pigments expressed on a chlorophyll basis (V+A+Z; mmol mol^−1^ Chl), (**b**) the de-epoxidation state (A+Z/V+A+Z), and (**c**) the total zeaxanthin are expressed on a chlorophyll basis (Z; mmol mol^−1^ Chl). Black and white rectangles on the *x*-axis symbolize subjective night and day, respectively (that is, when leaves would have experienced nighttime or daytime conditions). Time zero indicates the time when constant conditions started. Temporal patterns were examined with the generalized additive model (GAM) fitted with an automated smoothness selection [31]. Shaded areas indicate the 95% CI of the fitted GAM. Values are the mean ± S.E (*n* = 10).

**Figure 4:**
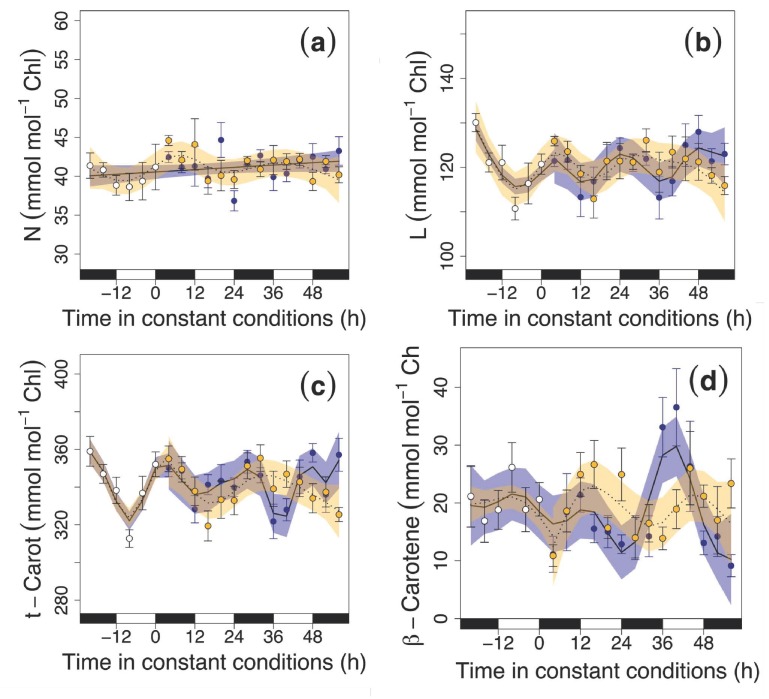
Carotenoids in rocket leaves during the last cycle of the entrainment phase (white circles) and under continuous darkness (blue circles) and continuous illumination conditions light (yellow circles): (**a**) neoxanthin expressed on a chlorophyll basis (N; mmol mol^−1^ Chl), lutein expressed on a chlorophyll basis (L; mmol mol^−1^ Chl), (**b**) total carotenoids expressed on a chlorophyll basis (t-Carot; mmol mol^−1^ Chl), (**c**) and β-carotene expressed on a chlorophyll basis (β-Car; mmol mol^−1^ Chl), and (**d**). Black and white rectangles on the *x*-axis represent subjective night and day, respectively (that is, when leaves would have experienced nighttime or daytime conditions). Time zero represents the time when constant conditions started. Temporal patterns were examined with the generalized additive model (GAM) fitted with an automated smoothness selection [31]. Shaded areas indicate the 95% CI of the fitted GAM. Values are the mean of 10 replicates ± S.E.

**Figure 5:**
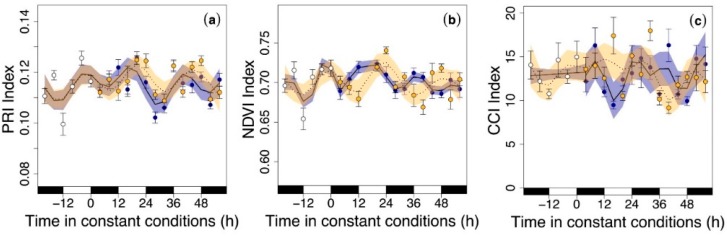
Optical indices in rocket leaves during the last cycle of the entrainment phase (white circles) and under continuous darkness (blue circles) and continuous illumination conditions light (yellow circles): (**a**) the photochemical reflectance index (PRI Index), (**b**) the normalized difference vegetation index (NDVI Index), and (**c**) the chlorophyll content index (CCI Index). Black and white rectangles on the *x*-axis represent subjective night and day, respectively (that is, when leaves would have experienced nighttime or daytime conditions). Time zero represents the time when constant conditions started. Temporal patterns were examined with the generalized additive model (GAM) fitted with automated smoothness selection [31]. Shaded areas indicate the 95% CI of the fitted GAM. Values are the mean of 16 replicates ± S.E.

**Figure 6:**
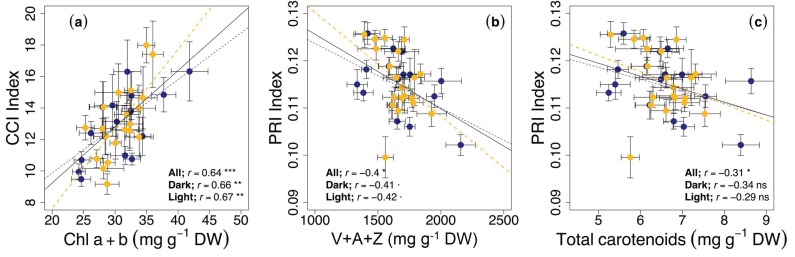
Relationships between (**a**) the chlorophyll content index (CCI Index) and total chlorophylls expressed on dry weight (Chl a+b; mg g^−1^ DW), (**b**)The photochemical reflectance index (PRI Index) and the total pool of xanthophyll cycle pigments expressed on a dry weight (mg g^−1^ DW), and (**c**) the PRI Index and total carotenoids expressed on a dry weight (mg g^−1^ DW), in rocket leaves during the last cycle of the entrainment phase and under continuous dark and light conditions. Values are the mean of 10 to 16 replicates ± S.E. Linear regressions are shown when significant at *p* < 0.05 (* *p* < 0.05; ** *p* < 0.001; *** *p* < 0.0001; n.s., not significant).

Differences among treatments were tested with one-way ANOVA and the Tukey post-hoc test (Figure 1). We examined temporal patterns of pigments and indices with the generalized additive model (GAM) fitted with an automated smoothness selection (10–15 nodes) [31]. We used a line of best fit from model predictions to estimate the extent of the diurnal oscillation (maximum minus minimum) during both the entrainment and constant phases. GAM models were statistically significant at α = 0.05 (Figures 2–5). Correlations were then tested to determine the relation between CCI and total chlorophyll, PRI and VAZ and PRI and total carotenoids (Figures 1c and 6). We calculated the *p*-values (statistically significant at α = 0.05) and coefficients, which are indicated on the figure. This analysis was performed in the R software environment (mgcv library in R 3.1.2, The R Foundation for Statistical Computing, Vienna, Austria).

## 3. Results

### 3.1. Maintenance of the Physiological Status and Visual Quality

The effect of illumination conditions on pigment composition was analyzed in rocket leaves packaged in protected atmosphere bags by exposing each of the bags to light/dark cycles in an entrainment phase of 3 days followed by continuous conditions of light (low light intensity that aimed to mimic the supermarket environment that was set at PAR ≈ 6 μmol m^−2^ s^−1^), or darkness for 2 days. Constant dark and light conditions for 48 h in the continuous phase of the experiment resulted in decreases in visual quality (Figure 1a), together with a slight decrease in the Fv/Fm ratio (which indicates the physiological status of the leaf (Figure 1b) under continuous dark. This positive relationship between visual quality and the Fv/Fm ratio was analyzed, resulting in a significant linear regression (Figure 1c). This indicates that Fv/Fm may also be a good indication of the vegetables quality. However, despite the continuous conditions, the lowest values for Fv/Fm were close to 0.65, and visual quality was higher than 4, indicating an overall good physiological state of the rocket. We also analyzed the content of other phytochemicals (tocopherols, phytosterols, and fatty acids) at the beginning of the entrainment phase and after 48 h of continuous conditions (Table 1), but we found that only α-tocopherol increased after 48 h under constant conditions (light or dark).

### 3.2. Circadian Regulation of Pigments

We observed rhythmic oscillations, with a period of ~24 h, in several phytochemicals after transferring the leaves to continuous light or dark conditions (Figure 2, Figure 3, Figure 4 and Figure 5). More specifically, we observed an oscillation in the Chl a to b ratio under both constant conditions: constant light and constant darkness (Chl a/b; Figure 2a), peaking around the end of the subjective nights (when it would have been night in the entrainment stage). The total chlorophyll pool (Chl a+b) oscillated under continuous dark conditions, but not under constant light (Figure 2b). Indeed, Chl a+b peaked around the subjective nights and declined during subjective days (when it would have been noon during the entrainment phase), with the amplitude of the oscillation being slightly higher than during the entrainment period.

We did not find any consistent rhythmic oscillation under continuous conditions in the total V+A+Z pool (Figure 3a) either during the dark or the light periods. However, there was a marked oscillation in the de-epoxidation state (A+Z/V+A+Z) (Figure 3b) and in Z on a chlorophyll basis (Z; Figure 3c) in rocket leaves under continuous illumination or continuous darkness. This indicates that circadian rhythms did not affect V+A+Z content but caused an oscillation in the de-epoxidation state, leading to a continuous change in Z. Interestingly, constant darkness enhanced the Z content after 42 h of darkness exposure as indicated by the largeness of the fluctuation, which was larger than during the entrainment period. Indeed, during entrainment, the de-epoxidation state and Z ranged from 0.045 mmol mol^−1^ Chl and 1.5 mmol mol^−1^ Chl, respectively, during the night up to a maximum of 0.07 and 2.5 mmol mol^−1^ Chl, respectively, at noon (estimated from the GAM best-fit line). During the constant light phase, A+Z/V+A+Z oscillated from 0.04 to 0.07 mol mol^−1^ and Z from 1 to 2.8 mmol mol^−1^ Chl. Therefore, the oscillation observed during constant light was 20% higher for A+Z/V+A+Z and 80% higher for Z than the same parameters under entrainment.

During 48 h of dark exposure, no oscillation was recorded in the N content (Figure 4a). However, we detected an oscillation in N (during light conditions), L (Figure 4b; both conditions), total carotenoids (t-Carot; Figure 4c; both conditions) and β-carotene content (β-Car; Figure 4d; both conditions), with these phytochemicals being affected by circadian rhythms and illumination conditions. Interestingly, for β-Car, this oscillation had an amplitude of 20 mmol mol^−1^ Chl, which was double the value measured in the entrainment phase (10 mmol mol^−1^ Chl), and this peak in β-Car content occurred after 42 h of continuous dark conditions.

### 3.3. Circadian Regulation of Reflectance Indices

To test whether pigment changes could be inferred from spectrometric indices, we analyzed the PRI (Figure 5a), the NDVI (Figure 5b) and CCI (Figure 5c) along with phytochemical analyses. We observed that the PRI, NDVI, and CCI oscillated with a frequency close to 24 h, suggesting that they were regulated by circadian rhythms. The only exception was the CCI during entrainment, when no oscillation was observed (Figure 5c).

As a consequence of the parallel oscillation under constant conditions, tight correlations between these indices and phytochemicals were observed. In fact, the correlation of CCI with total Chl a+b (Figure 6a) was significantly positive, and the correlations of the PRI with V+A+Z pigments (Figure 6b) and total carotenoids (Figure 6c) were significantly negative.

## 4. Discussion

Circadian rhythms permit living organisms to anticipate, adapt, and respond early to predictable daily environmental changes, optimizing their physiology, metabolism, behavior, and adjusting their biology, accordingly [32]. Overall, the circadian clock is an important driver of general fitness and photosynthesis in plants [13,32]. Recently, it was reported that the circadian clock translates the oscillation to photosynthetic pigments, including chlorophylls and carotenoids [15,33]. Here we demonstrated that chlorophylls and carotenoids of rocket leaves stored in commercial protected bags for human consumption are also under circadian regulation, but this fact is not considered in the storage procedures employed at either the post-harvest or supermarkets stages.

To study the occurrence of rhythms, we entrained rocket leaves under light/darkness cycles of 12/12 h for 3 days, and then we examined free-run oscillations under 2 days of constant light or constant darkness. In agreement with the rhythmic changes of Chl a/b observed in entire plants [15], Chl a/b oscillated in both constant conditions (Figure 2a) in packaged rocket leaves. However, we only detected oscillations of total Chl a+b under darkness conditions (Figure 2b). Chlorophylls are responsible for the green color of leaves and, consequently, an increase in Chl a+b is synonymous with better visual quality (more greening). Continuous light conditions, as applied here to emulate the practice of some supermarkets (especially those open 24 h), may cause photobleaching of chlorophylls and even lethal injuries [34]. We reported that total Chl a+b peaked around subjective nights and declined during subjective days (Figure 2). This means that rocket leaves avoid the physiological damage that occurs when vegetables are exposed to excessive light by anticipating day-night cycles.

We observed ~24 h oscillations in the production of different phytochemicals (β-Car, L and Z) during the continuous light and dark periods (Figure 2 and Figure 3). Zeaxanthin and β-Car peaked during subjective days, and their concentrations declined during the subjective nights. The increase in the ratio A+Z/V+A+Z (Figure 3b) indicated Z formation during of the V+A+Z cycle due to the activity of the enzymes involved in the cycle, which have been shown to be under circadian regulation [35]. We did not find a circadian rhythm in the total xanthophyll cycle (Figure 3a) due to the interconversion of the xanthophylls of the cycle, which keeps the total pool stable. There were also oscillations in L and in total carotenoid content under constant conditions, but the width of the fluctuation was slightly lower than during the entrainment period, which led to a slight decrease in their concentrations. Daily peaks in the circadian regulation of carotenoid biosynthesis genes have been reported [36], which may explain the circadian patterns found here. Indeed, the pattern observed with the maximum peaks occurring at subjective noon may be related to a plant strategy for photodamage avoidance (Figure 3d and Figure 4d). We did not observe pigment changes in N (Figure 4a) that is in connection with the fact that N is considered structural carotenoid with low change under environmental cues [37]. In general, it is thus tempting to hypothesize that being able to maintain circadian rhythmicity is one of the processes that aids maintenance of a healthy physiological state and good visual quality of rocket during storage (Figure 1, Figure 2, Figure 3, Figure 4 and Figure 5). In this sense, circadian regulation should be taken into account as an important cue for phytochemical content and to preserve the nutritional content of plant-derived food [16].

The intensity of the light is also an important factor in the pigment response. Indeed, low growth light supplemented with several short daily light pulses of higher intensity increases the carotenoid Z in *Arabidopsis thaliana* [38]. Here we demonstrate that even under low light intensities that aim to mimic the environment in the supermarkets (≈6 μmol m^−2^ s^−1^) we found that carotenoids were light responsive. Overall, our results indicate that rocket stored in supermarket shelves maintains circadian rhythms that regulate photosynthetic pigments. It is thus to be expected that the nutritional quality of the green leaves may change daily or even hourly, therefore, supermarket storage policies should take these factors into account.

Chromatographic techniques employed to determine pigment composition are accurate but also time-consuming and expensive. Alternatively, it is possible to estimate pigment contents using non-destructive and in situ optical techniques, such as the PRI, NDVI (reflectance-based indices) and CCI (transmittance-based index). Our results show that the PRI and NDVI oscillated with a frequency close to 24 h, peaking during subjective days and noon, respectively, with an oscillation amplitude similar to that reported during the entrainment phase. This is the first time, to our knowledge, that circadian oscillations in these indices have been reported. Consequently, we can recommend the use of these indices as a non-destructive way to monitor the pigment pool in supermarkets to maintain high nutritional quality.

Although the three indices showed a reasonable performance, the CCI showed the opposite oscillation during constant light and dark conditions, peaking during subjective days and nights, respectively. We report a significant and high correlation of reflectance indices with carotenoid and chlorophyll contents (Figure 6). To the best of our knowledge, this is also the first test of the capacity of these indices to function as nutraceutical reporters in the food quality area. Further research is needed to develop equations to interconvert optical measurements to relative nutraceutical (carotenoid) content.

## 5. Conclusions

Fresh vegetables stored on supermarket shelves are under circadian regulation provided that some external factor, such as light, activates their rhythms. Thus, the present study demonstrates that both phytochemicals and reflectance-based indices are under circadian regulation, resulting in an oscillation of the nutritional quality of stored vegetables. Overall, given the importance of preserving food quality, the existence of such internal rhythms during continuous conditions opens new horizons in nutrition science, supermarket managements, and household storage. The proposed method here to estimate the nutritional quality is promising for nutritional quality assessments. In addition, this technique could also be used in urban vertical-farms (growing crops in layers above one another under climate-controlled environments) to enhance productivity and decrease their footprint [39], or even in space stations (where phytochemical production and nutritional content are the final goals).

## Figures and Tables

**Table 1:** Tocopherol (Tf) (mg/100 g DW) and phytosterol (mg/100 g DW) contents and the percentage of fatty acids with respect to the total fatty acids at −20 h and 48 h from the beginning of the continuous phase.

**Tocopherol Content (Tf) (mg/100 g DW)**											
**Time in Constant Conditions (Hours)**	**α-Tf**			**β-Tf**	**γ-Tf**			**δ-Tf**	**Total Pool**		
−20 h	8.41 ± 0.4			-	-			-	8.41 ± 0.4		
48 h (dark)	12.29 ± 2.0			-	-			-	12.29 ± 2.0		
48 h (light)	12.29			-	-			-	12.29		
**Phytosterol Content (mg/100 g DW)**											
**Time in Constant Conditions (hours)**	**Δ^5^-Avenasterol**			**Campesterol**			**β-Sitoesterol**			**Total Pool**	
−20 h	32.26 ± 3.6			16.13 ± 3.1			66.45 ± 5.7			114.84	
48 h (dark)	30.86 ± 9.7			14.84 ± 4.8			52.39 ± 7.25			98.09 ± 2.3	
48 h (light)	22.04 ± 13.2			11.18 ± 2.1			57.45 ± 15.7			90.67 ± 3.3	
**% of Fatty Acids with Respect to the Total**											
**Time in Constant Conditions (hours)**	**Palmitic Acid (16:0)**				**Linoleic Acid (18:2*n*6)**				**Linolenic Acid (18:3*n*3)**		
−20 h	16.9 ± 0.2				15.5 ± 0.0				67.3 ± 0.2		
40 h (dark)	16.9 ± 0.2				15.9 ± 0.0				67.1 ± 0.2		
40 h (light)	18.0 ± 0.1				14.7 ± 0.1				67.2 ± 0.2		
